# The metalloprotein YhcH is an anomerase providing N-acetylneuraminate aldolase with the open form of its substrate

**DOI:** 10.1016/j.jbc.2021.100699

**Published:** 2021-04-23

**Authors:** Takfarinas Kentache, Leopold Thabault, Gladys Deumer, Vincent Haufroid, Raphaël Frédérick, Carole L. Linster, Alessio Peracchi, Maria Veiga-da-Cunha, Guido T. Bommer, Emile Van Schaftingen

**Affiliations:** 1 Laboratory of Physiological Chemistry, de Duve Institute, UCLouvain, Brussels, Belgium; 2 Medicinal Chemistry Research Group (CMFA), Louvain Drug Research Institute (LDRI), UCLouvain, Brussels, Belgium; 3 Louvain Center for Toxicology and Applied Pharmacology, UCLouvain, Brussels, Belgium; 4 Luxembourg Centre for Systems Biomedicine, University of Luxembourg, Belvaux, Luxembourg; 5 Department of Chemistry, Life Sciences and Environmental Sustainability, University of Parma, Parma, Italy; 6 Walloon Excellence in Life Sciences and Biotechnology (WELBIO), UCLouvain, Brussels, Belgium

**Keywords:** aldolase, anomerase, sialic acid, mutarotase, metalloprotein, 2-KDXyl, 2-keto-3-deoxyxylonate, 2,3-EN, 2,3-dehydro-2-deoxy-N-acetylneuraminate, 2,7-AN, 2,7-anhydro-Neu5Ac, KDN, 2-keto-3-deoxynonanoate, NanA, *E. coli* Neu5Ac aldolase, Neu5Ac, N-acetylneuraminate, NPL, human Neu5Ac aldolase, TMSP, 2,2,3,3-^2^H_4_ (trimethylsilyl)propionic acid sodium salt

## Abstract

N-acetylneuraminate (Neu5Ac), an abundant sugar present in glycans in vertebrates and some bacteria, can be used as an energy source by several prokaryotes, including *Escherichia coli*. In solution, more than 99% of Neu5Ac is in cyclic form (≈92% beta-anomer and ≈7% alpha-anomer), whereas <0.5% is in the open form. The aldolase that initiates Neu5Ac metabolism in *E. coli*, NanA, has been reported to act on the alpha-anomer. Surprisingly, when we performed this reaction at pH 6 to minimize spontaneous anomerization, we found NanA and its human homolog NPL preferentially metabolize the open form of this substrate. We tested whether the *E. coli* Neu5Ac anomerase NanM could promote turnover, finding it stimulated the utilization of both beta and alpha-anomers by NanA *in vitro*. However, NanM is localized in the periplasmic space and cannot facilitate Neu5Ac metabolism by NanA in the cytoplasm *in vivo*. We discovered that YhcH, a cytoplasmic protein encoded by many Neu5Ac catabolic operons and belonging to a protein family of unknown function (DUF386), also facilitated Neu5Ac utilization by NanA and NPL and displayed Neu5Ac anomerase activity *in vitro*. YhcH contains Zn, and its accelerating effect on the aldolase reaction was inhibited by metal chelators. Remarkably, several transition metals accelerated Neu5Ac anomerization in the absence of enzyme. Experiments with *E. coli* mutants indicated that YhcH expression provides a selective advantage for growth on Neu5Ac. In conclusion, YhcH plays the unprecedented role of providing an aldolase with the preferred unstable open form of its substrate.

Most of the enzymes of the classical metabolic pathways are molecularly identified. Yet even in the case of widely studied organisms, such as humans, *Saccharomyces cerevisiae*, and *Escherichia coli*, there seem to be still many putative enzymes whose molecular function has not yet been identified (1). Enzymes that are likely to have been understudied (to the major exception of carbonic anhydrases) are those that accelerate spontaneous reactions. Major reasons for this oversight probably include that such enzymes may seem to be unnecessary and that they are not essential for survival under standard conditions. Furthermore, the background “activity” of the spontaneous reaction makes them difficult to investigate. The present paper is devoted to the identification of a new example of such an enzyme, catalyzing the ring opening of the pyranose form of a sugar to facilitate its metabolism by an aldolase.

Sialic acids form a large family of nine carbon sugar acids, the most common of which is N-acetylneuraminic acid (Neu5Ac). Sialic acids are most often found at the extremities of glycans present on extracellular proteins and at the surface of vertebrate cells, where they serve for diverse interactions. Sialoconjugates are particularly abundant in the gastrointestinal and respiratory tracts, where they may interact with bacteria. Many of these bacteria have acquired the capacity to metabolize sialoconjugates and/or sialic acids. Some of them, such as *E. coli* (2) and *Ruminococcus gnavus* (3), can grow on Neu5Ac and other related compounds as the only energy-providing substrate.

The first enzymatic step in the degradation of sialic acid is its cleavage by N-acetylneuraminate lyase, an enzyme catalyzing an aldolase type of reaction leading to pyruvate and N-acetylmannosamine formation (4). The latter is then phosphorylated to N-acetylmannosamine-6-phosphate, which is further metabolized by an epimerase. N-acetylneuraminate is metabolized by a similar pathway in mammalian cells (5).

The enzymes and the transporters involved in sialic acid metabolism in bacteria are usually encoded by operons. The role of most of the enzymes encoded by these operons is now identified (references including (6, 7, 8)), with the notable exception of YhcH, a protein that is almost always found in bacteria possessing Neu5Ac aldolase and is frequently encoded by the same operon (9). YhcH belongs to the DUF386 or YhcH/YjgK/YiaL family, whose members are found in Gram-negative and Gram-positive bacteria, but not in archaea or eukaryotes. No precise function is known for any member of the DUF386 family. *E. coli* strains deficient in YhcH reportedly do not show a growth defect on sialic acid (10). In the context of a wide structural genomic project, *Haemophilus influenzae* YhcH was crystallized and its structure determined (11). The putative active site contains a copper ion coordinated to four amino acid side chains. The conclusion of this work, based mainly on the operon context of closely related YhcHs, was that this protein might be a sugar isomerase involved in the processing of exogenous sialic acid by possibly catalyzing an epimerase reaction. However, no experimental support was provided for this speculation.

In solution, more than 99% of Neu5Ac is in a cyclic form, mainly the beta-anomer (91–95%), but also the alpha-anomer (≈5–9%), while <0.5% is in an open form (12, 13, 14, 15). Many enzymes that act on sugars show preference for one anomer, and some evidence supports that Neu5Ac aldolase acts on the alpha-anomer (12, 16, 17). This preference is puzzling, as the reaction mechanism involves opening of the substrate to form a Schiff’s base with a lysine in the active site (18).

As described in the present article, we recently reinvestigated this problem with the help of the Neu5Ac anomerase NanM and concluded that *in vitro*, this anomerase greatly facilitates the reaction catalyzed by Neu5Ac aldolase by providing this enzyme with the open form of its substrate. As NanM is physiologically present in the periplasmic space, we wondered whether a cytosolic protein of as yet unknown function would catalyze this reaction and found that YhcH acts as an anomerase that opens the cyclic forms of Neu5Ac, thereby facilitating its utilization by the aldolase. This role of YhcH is the first example of an anomerase serving physiologically to provide another enzyme with an open sugar.

## Results

### The true substrate of Neu5Ac aldolase is the open sugar

**Figure 1:**
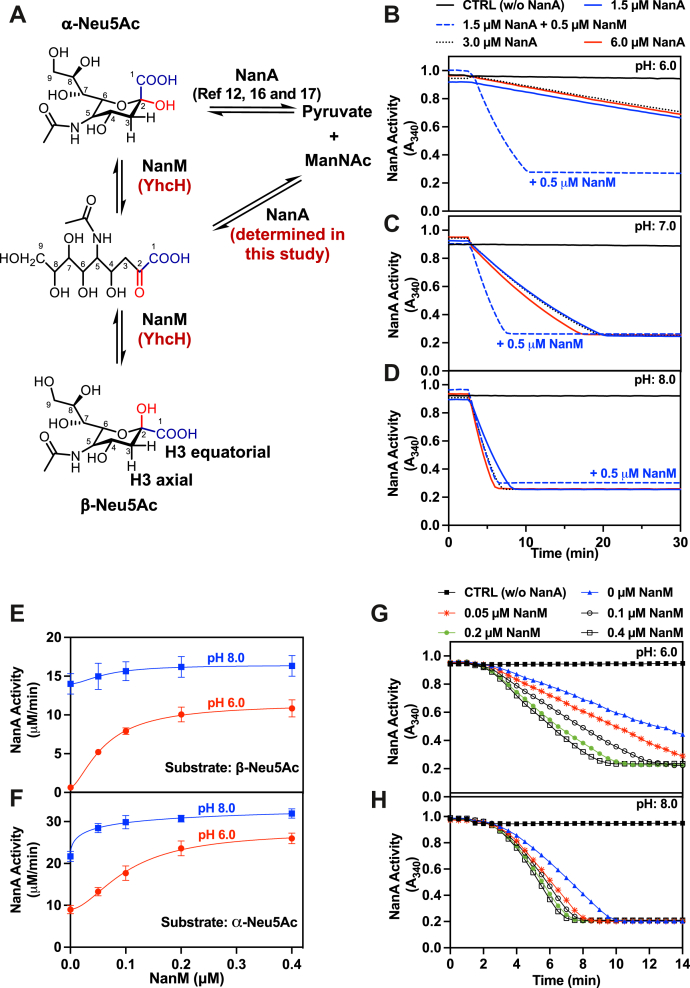
**Evidence that the Neu5Ac pyruvate aldolase NanA utilizes the open form of Neu5Ac.***A*, schematic representation of the various forms of Neu5Ac and the reactions catalyzed by the anomerase NanM and YhcH. The Neu5Ac aldolase NanA, which was previously thought to use the alpha-anomer, is now shown to use the open form of Neu5Ac. *B*–*D*, spectrophotometer tracings of Neu5Ac aldolase activity illustrating the effect of different concentrations of the aldolase NanA (1.5, 3.0, and 6.0 μM) and of the anomerase NanM at pH 6.0 (*B*), 7.0 (*C*), and 8.0 (*D*). NanM was added at 0.5 μM in some assays containing 1.5 μM NanA. Neu5Ac was present at 200 μM and NADH at 150 μM. The assays were conducted at 20 °C. *E* and *F*, effect of NanM to stimulate the utilization of α-Neu5Ac by NanA. Spectrophotometer tracings with 2 μM NanA and different concentrations of NanM at pH 6.0 (*E*) and 8.0 (*F*). At pH 6.0, α-Neu5Ac was generated from 3’ sialyllactose in the presence of 0.6 U/ml sialidase A. At pH 8.0, α-Neu5Ac was generated from 2,7-AN in the presence of 3 μM YjhC and 50 μM NAD^+^. The assays were conducted at 20 °C. *G* and *H*, effect of the concentration of NanM on the activity of NanA acting on either β-Neu5Ac (*G*) or α-Neu5Ac (*H*). The assays were conducted at 20 °C, at pH 6.0 or pH 8.0, with the indicated concentrations of NanM. NanA was added at 2 μM. Substrate concentration was 120 μM for Neu5Ac and 1 mM for 2,7-AN and 3’-sialyllactose. Results shown are means ± SD (n = 3).

Solutions of Neu5Ac contain about 7% of the alpha-anomer of sialic acid (α-Neu5Ac) (Fig. 1*A*) (12, 15). To verify that α-Neu5Ac is the true substrate of Neu5Ac aldolase (NanA), we used a spectrophotometric assay in which the pyruvate formed by this enzyme is reduced by lactate dehydrogenase to L-lactate with concomitant consumption of NADH (6) for the assay. These experiments were initially performed at pH 6, where spontaneous anomerization is slow (19). Adding *E. coli* recombinant NanA to such an assay containing 200 μM Neu5Ac did not lead to the expected rapid consumption of ≈14 μM NADH (ΔA_340_ = 0.084 AU), if α-Neu5Ac was rapidly consumed by the aldolase (Fig. 1*B*). Instead, a slow NADH consumption was observed, that was very similar at the three different NanA concentrations. This excluded both alpha and beta-anomer as primary substrate and indicated that the rate-limiting step in the assay was not the aldolase activity, but rather the supply of the true substrate to this enzyme, likely by spontaneous conversion of the beta and alpha-anomers to the linear form. Accordingly, the NADH consumption was about five times faster at pH 7 and even more so at pH 8, in agreement with the well-known effect of alkalinization on the spontaneous anomerization of Neu5Ac (19) (Fig. 1, *C* and *D*).

Remarkably, the addition of the anomerase NanM accelerated more than 20-fold the rate of the reaction at pH 6 (Fig. 1*B*), to a lesser extent at pH 7 (Fig. 1*C*), and minimally at pH 8.0 (Fig. 1*D*). Of note, the concentration of NanM (0.05 μM) needed to stimulate the reaction half-maximally was about 40-fold lower than the concentration (2 μM) of the aldolase NanA (Fig. 1*E*), indicating that the effect observed is not due to the formation of a complex between NanA and NanM. Taken together these findings hinted that the real substrate of Neu5Ac aldolase is neither the beta-anomer, nor the alpha-anomer, but most likely the linear form of Neu5Ac.

NanM interconverts the alpha and the beta-anomers *via* the open form. Therefore, its stimulatory effect on the activity of the aldolase (NanA) in a solution containing mostly the beta-anomer could be due either to the alpha-anomer or to the linear form being the substrate for NanA. To distinguish between these two possibilities, we performed experiments in which the alpha-anomer is generated in the assay cuvette by hydrolysis of a sialylconjugate by *Clostridium* sialidase A (12). If the alpha-anomer is the true substrate, one would expect that addition of the anomerase would decrease the rate of the aldolase reaction, since it would reduce the concentration of alpha-anomer by converting it into the linear form and the beta-anomer. Yet, as shown in Figure 1, *F*–*H*, the opposite was observed: addition of the anomerase NanM accelerated the reaction up to ≈4 fold at pH 6, further indicating that the open form is the real substrate and that the anomerase allows its rapid reformation from the alpha-anomer as soon as it is consumed by the aldolase.

### YhcH behaves as an anomerase facilitating the reaction of Neu5Ac aldolase

Neu5Ac is transported by the carrier NanT as its beta-anomer across the inner membrane of *E. coli* and NanM is present in the periplasmic space to facilitate the utilization of the alpha-anomer (20). Due to its location in the periplasmic space, NanM cannot provide the aldolase, a cytoplasmic enzyme, with the linear form of Neu5Ac. Therefore, we hypothesized that another enzyme might perform this function in the cytoplasm.

**Figure 2:**
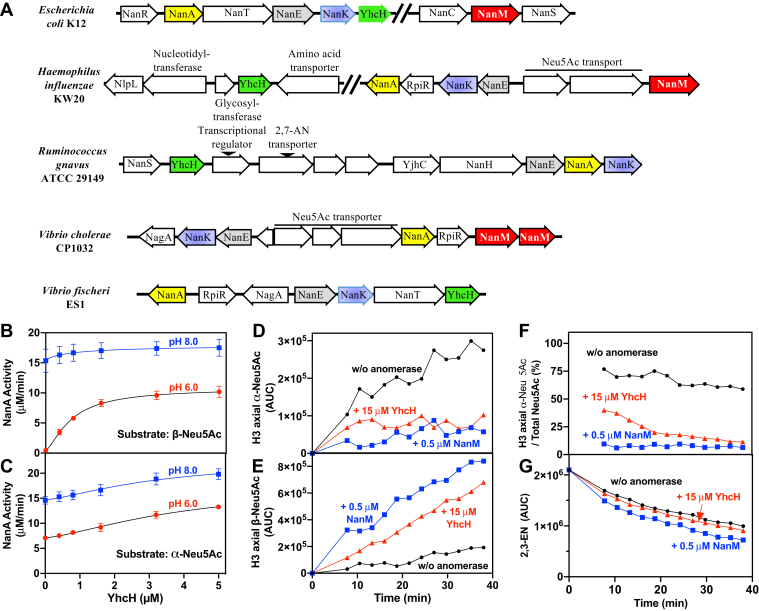
**YhcH encoded by Neu5Ac catabolic operons is an anomerase that facilitates the metabolism of Neu5Ac by Neu5Ac aldolase.***A*, organization of genomic regions harboring YhcH (*green arrow*) and NanM (*red arrow*) in *Escherichia coli* K12, *Haemophilus influenzae* KW20, *Ruminococcus gnavus* ATCC 29149, *Vibrio cholerae* 01, and *Vibrio fischeri* ES1. *B* and *C*, as shown in Fig. 1, *G* and *H* for NanM, YhcH stimulates the utilization of both β- and α-Neu5Ac by NanA. Results shown are means ± SD (n = 3). *D*–*G*, ^1^H-NMR analysis comparing the anomerase activity of YhcH and NanM. 2,3-EN (1 mM) was incubated at 10 °C in 50 mM deuterated acetate buffer, pH 6, with 20 μM NAD^+^, 1.5 μM YjhC (5), in the absence (in *black*) or the presence of 0.5 μM NanM (in *blue*) or 15 μM YhcH (in *red*). Repetitive NMR spectra were taken at different times following the initiation of the reaction by addition of YjhC. The α (*D*) and β (*E*) forms of Neu5Ac were computed from the shift of axial H3 at 1.65 and 1.8 ppm, respectively. The proportion of α-Neu5Ac in the total pool of Neu5Ac was computed from these values (*F*). The amount of 2,3-EN shown in (*G*) was calculated from the shift of N5-H in the 8 ppm region. AUC, area under the curve.

We focused our attention on YhcH, a protein of unknown function encoded by sialic acid operons (Fig. 2*A*). Producing recombinant YhcH with a His-tag at the N-terminus (as in the ASKA collection (21)) did not provide satisfactory yield, presumably because the N-terminus of the protein interacts with the neighboring subunit in the dimer as in the structure of the *H. influenzae* homolog (11). Thus, the recombinant protein was produced with a C-terminal His-tag. Like the anomerase NanM, recombinant *E. coli* YhcH increased the rate of the aldolase reaction when a solution of Neu5Ac (essentially the beta-anomer) was used as a substrate. However, we noted that about tenfold higher concentrations of YhcH than of NanM were required to reach a similar effect (Fig. 2, *B* and *C*). We also noted that YhcH stimulated the activity when the alpha-anomer of Neu5Ac was produced. However, to observe this effect, we had to use higher concentrations of YhcH than required to obtain the effect on the beta-anomer.

These findings suggested that YhcH is able to catalyze a full anomerase reaction, though less efficiently than NanM. This was confirmed by using NMR to follow the formation of the beta-anomer of Neu5Ac from the alpha-anomer, as generated by the NAD^+^-dependent hydratase YjhC from 2,3-dehydro-2-deoxy-N-acetylneuraminate (2,3-EN) (6). The alpha-anomer (Fig. 2*D*) and the beta-anomer (Fig. 2*E*) (each assessed with the shift corresponding to axial H3) appeared progressively in the absence of YhcH or NanM. The presence of 15 μM YhcH and even more so of 0.5 μM NanM reduced the accumulation of the alpha-anomer (Fig. 2*D*) and stimulated the appearance of the beta-anomer (Fig. 2*E*), thus strongly decreasing the ratio of alpha/beta anomers (Fig. 2*F*). Both anomerases modestly stimulated the utilization of 2,3-EN and lowered the formation of 2,7-AN (Fig. S1), presumably by reducing the inhibition exerted by α-Neu5Ac on YjhC (6). These experiments demonstrated that YhcH catalyzes a full anomerase reaction, though less efficiently than NanM. Furthermore, the conversion of the beta-anomer to the linear form by YhcH was more efficient than the conversion of the alpha-anomer to the open form.

### Further evidence that NanM and YhcH do not activate Neu5Ac aldolase but provide the linear form of substrate

**Figure 3:**
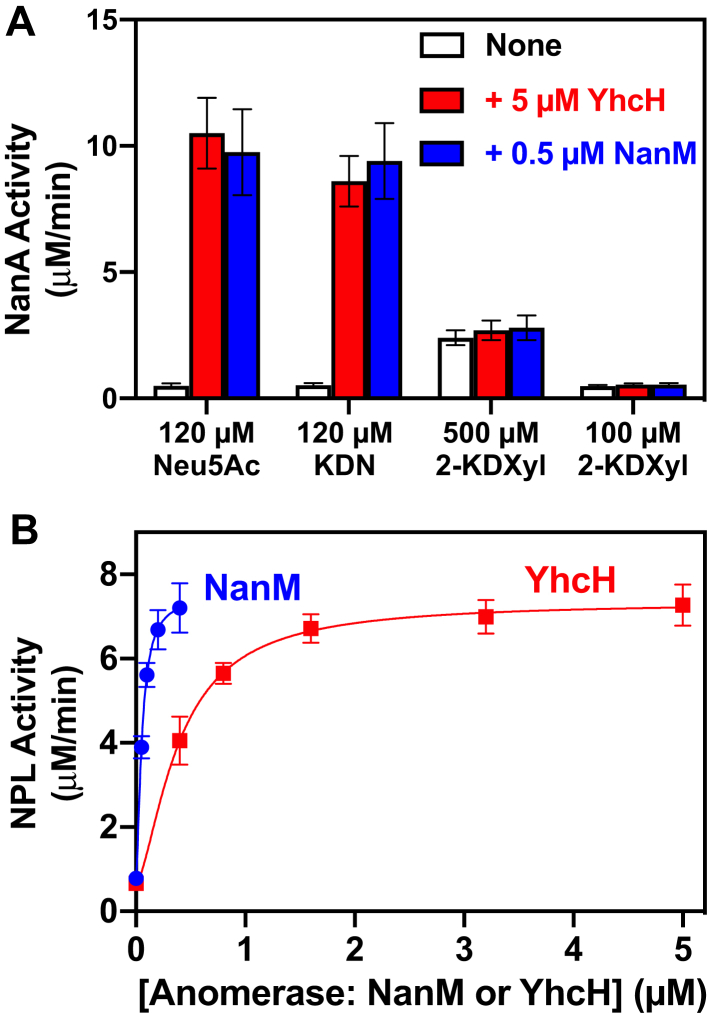
**The anomerases YhcH and NanM do not act through a stimulation of the aldolase activity.***A*, NanM and YhcH increase the aldolase activity of NanA on two substrates, Neu5Ac and KDN, which are essentially in cyclic form, but not on the smaller substrate, 2-keto-3-deoxyxylonate (2-KDXyl), which is largely in the open form. Assays were conducted at pH 6.0 and 20 °C with 0.5 μM NanM or 5 μM YhcH. NanA was added at 2 μM. Neu5Ac, KDN, or 2-KDXyl was used at the indicated concentrations. *B*, the effect of *E. coli* YhcH and NanM to facilitate Neu5Ac utilization is also observed with human Neu5Ac aldolase NPL. Assays were carried out at pH 6.0 and 20 °C in the presence of the indicated concentrations of NanM and YhcH. The concentrations of NPL and Neu5Ac were 6 μM and 120 μM, respectively. Results shown are means ± SD (n = 3).

We considered the possibility that NanM and YhcH acted by directly stimulating the activity of Neu5Ac aldolase rather than by providing the appropriate substrate. To distinguish between these two possibilities, we used 2-keto-3-deoxyxylonate. This compound is a weak substrate of Neu5Ac aldolase, but, unlike Neu5Ac, it is largely present in the open form (about 30%; supplementary data in ref (22)). If YhcH and NanM stimulated the enzymatic activity of NanA by interacting with it, this should also accelerate the reaction of NanA on 2-keto-3-deoxyxylonate. Yet, no such effect was observed (Fig. 3*A*), supporting the conclusion that the anomerases act by providing the open substrate. In contrast, both anomerases stimulated the conversion of 2-keto-3-deoxynonanoate (KDN), the deamino analogue of Neu5Ac, which is also essentially present in cyclic forms. Of note, the latter observation also indicates that the N-acetylamino group of Neu5Ac is not essential for substrates of the anomerases NanM and YhcH.

To provide further support to the idea that NanM and YhcH do not act by stimulating the activity of NanA, we tested the effect of these *E. coli* anomerases on the human homolog of NanA (NPL), which only shares about 30% sequence identity with the *E. coli* NanA. This low degree of identity and the absence of a YhcH homolog in eukaryotes make it unlikely that the human NPL would have “conserved” an ancestral binding site present in its prokaryotic homologs. We noted that both NanM and YhcH stimulated the activity of human NPL on Neu5Ac (Fig. 3*B*), further arguing in favor of the idea that they act by providing the appropriate form of the substrate.

To confirm that NanM and YhcH can indeed provide a linear form of Neu5Ac, we took advantage of the fact that the keto group in the linear Neu5Ac can form a semicarbazone when reacting with semicarbazide (23). Incubation with semicarbazide and measurement of the absorbance at 249 nm indicated that the formation of semicarbazone was stimulated up to about threefold by NanM (Fig. S2). In the case of YhcH, the acceleration was only observed at very low concentrations of semicarbazide, presumably because semicarbazide inhibited the anomerase activity. The reaction of semicarbazide with 2-keto-3-deoxyxylonate, the sialic acid analog that is 30% in its open keto form (see above) was about 180-fold faster than the reaction with sialic acid. As expected, NanM or YhcH did not have any effect on the rate of semicarbazone formation from the largely linear compound 2-keto-3-deoxyxylonate (Fig. S2). Altogether, these findings indicate that the anomerases NanM or YhcH facilitate the action of Neu5Ac aldolase by producing the open form of substrate.

### YhcH contains Zn, and some metal cations have an intrinsic anomerase activity

Previous data have shown that *H. influenzae* YhcH, which shares about 60% sequence identity with *E. coli* YhcH, contains a tightly bound copper, presumably Cu^2+^, in its catalytic site (11). Therefore, we determined the metal content of purified *E. coli* YhcH, produced on a rich medium (LB) (Table S1). The protein mostly contained Zn (0.41 mol/mol) and lower amounts of Cu (0.051 mol/mol), Ni (0.061 mol/mol), and Mn (0.021 mol/mol). No significant amount of metal was found to be associated with NanM or NanA. We tried to enrich recombinant YhcH with different metal supplements in the culture medium. Enrichment with copper (80 μM CuCl_2_) did not lead to a significant increase in the Cu content (0.055 mol/mol) or decrease in the Zn content (0.467 mol/mol), while addition of MnCl_2_ (60 μM) led to an increase of Mn content (to 0.873 mol/mol) and a decrease in Zn content (0.308 mol/mol). Enrichment with ZnCl_2_ (30 μM) led to an increase in the Zn content to 1.13 mol/mol. Because of the preference of the protein for Zn, the Zn-enriched YhcH was used for all enzymatic studies. We also produced recombinant *H. influenzae* YhcH in *E. coli* grown on nonmetal-enriched LB medium. The metal content was 0.89, 0.014, 0.019, and 0.052 mol/mol for Zn, Cu, Ni, and Mn, respectively. Thus, the *H. influenzae* YhcH also showed a marked preference for Zn, similar to the *E. coli* protein. We have no explanation for the contradiction with previous results (11). We also tested the ability of *H. influenzae* YhcH to stimulate the activity of NanA on Neu5Ac and found that as expected it behaved like *E. coli* YhcH (not shown). This indicated that Zn can indeed be incorporated into functional YhcH.

**Figure 4:**
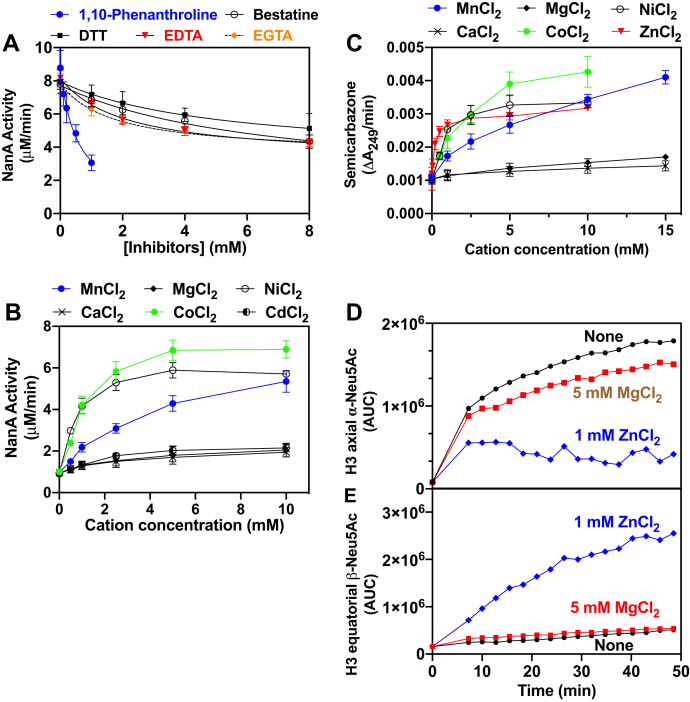
**YhcH is inhibited by metal chelators and its anomerase activity is mimicked by divalent cations of transition metals.***A*, inhibition of the YhcH activity by metal chelators. The Neu5Ac aldolase activity of *E. coli* NanA was tested at pH 6.0 and 20 °C in the presence of 200 μM Neu5Ac, 2 μM NanA, 0.8 μM YhcH, and the indicated concentrations of 1,10-phenanthroline, bestatin, DTT, EDTA, and EGTA. *B*, stimulation of the utilization of Neu5Ac by *E. coli* NanA by divalent cations. The Neu5Ac aldolase activity of *E. coli* NanA was tested at pH 6.0 and 20 °C in the presence of 120 μM Neu5Ac, 2 μM NanA, and the indicated concentrations of divalent cations. *C*, effect of divalent cations on the formation of a semicarbazone from Neu5Ac. The assay mixture contained 100 mM potassium phosphate, pH 6.0, 10 mM semicarbazide, and the indicated divalent cations. The assay was started by addition of 100 μM Neu5Ac and the formed semicarbazone-Neu5Ac complex was monitored spectrophotometrically at 249 nm. The assay was conducted at 20 °C. Results shown are means ± SD (n = 3). *D* and *E*, ^1^H-NMR assay demonstrating the effect of Zn^2+^ on Neu5Ac anomerization. 3’-Sialyllactose (1 mM) was incubated with *C. perfringens* sialidase (0.6 U/ml) with or without 1 mM ZnCl_2_ or 5 mM MgCl_2_ at 10 °C. The shifts corresponding to axial H3 of α-Neu5Ac (≈1.65 ppm) (*D*) and equatorial H3 of β-Neu5Ac (≈2.25 ppm) (*E*) are shown to illustrate the evolution of the alpha and beta-anomers. The other H3 shifts were not exploitable because of superimposition with shifts corresponding to 3’-sialyllactose. AUC, area under the curve

We also determined if metal chelators would decrease the activity of *E. coli* YhcH. Figure 4*A* shows that this was indeed the case, particularly with 1,10-phenanthroline. This effect was antagonized by adding ZnCl_2_ or MnCl_2_ (Fig. S3). While trying to supplement chelator-inhibited YhcH with divalent cations, we noted that some of them stimulated the NanA activity in the absence of the anomerases YhcH or NanM, which suggested that the divalent cations also catalyzed the anomerization of Neu5Ac. The effect of different divalent cations on the cleavage of Neu5Ac by NanA is illustrated in Figure 4*B*. Co^2+^, Ni^2+^, and Mn^2+^ stimulated the reaction by up to 5- to 7-fold while Cd^2+^, Mg^2+^, and Ca^2+^ had much lower, barely detectable effects. Zn^2+^ could not be tested because it markedly inhibited NanA and LDH activity at concentrations >10 μM. Mn^2+^ and Co^2+^ also stimulated the cleavage of KDN, but not of 2-keto-3-deoxyxylonate (not shown), indicating that they acted by enhancing the opening of the substrate Neu5Ac and KDN rather than as cofactors that would stimulate the aldolase NanA.

Further evidence that divalent cations accelerated the formation of the open form of Neu5Ac was obtained by showing that they accelerated the rate of the reaction of Neu5Ac with semicarbazide. The most potent cation was Zn^2+^, followed by Ni^2+^, Co^2+^, and Mn^2+^, while Ca^2+^ and Mg^2+^ were almost devoid of effect, as observed when this was determined with the help of the NanA activity (Fig. 4*C*). Cu^2+^, which was tested at a wavelength of 260 nm rather than 249 nm, had a similar effect as Co^2+^ (not shown).

To unequivocally demonstrate the anomerase activity of divalent cations, we used NMR to follow the conversion of the alpha-anomer of Neu5Ac to the beta-anomer in the presence and absence of Zn^2+^. In this experiment, we chose to generate α-Neu5Ac from 3’-sialyllactose with *Clostridium* sialidase A, rather than 2,3-EN and its hydratase YjhC (5), because the latter enzyme was found to be potently inhibited by Zn^2+^. As shown in Figure 4, *D* and *E*, Zn^2+^ markedly accelerated the formation of beta-anomer (as assessed by the shift of the beta H3eq) and the disappearance of the alpha-anomer (as assessed by the shift of the alpha H3ax). Mg^2+^ (Fig. 4, *D* and *E*) and Ca^2+^ had much smaller effects. Co^2+^ and Ni^2+^ also stimulated the appearance of β-Neu5Ac, but their effects were difficult to quantify due to broadening of the NMR signal (not shown). In contrast, we could not assess the effect of Mn^2+^ and Cu^2+^ due to the complete disappearance of the NMR signals.

### Inactivation of YhcH leads to decreased growth rate on Neu5Ac

**Figure 5:**
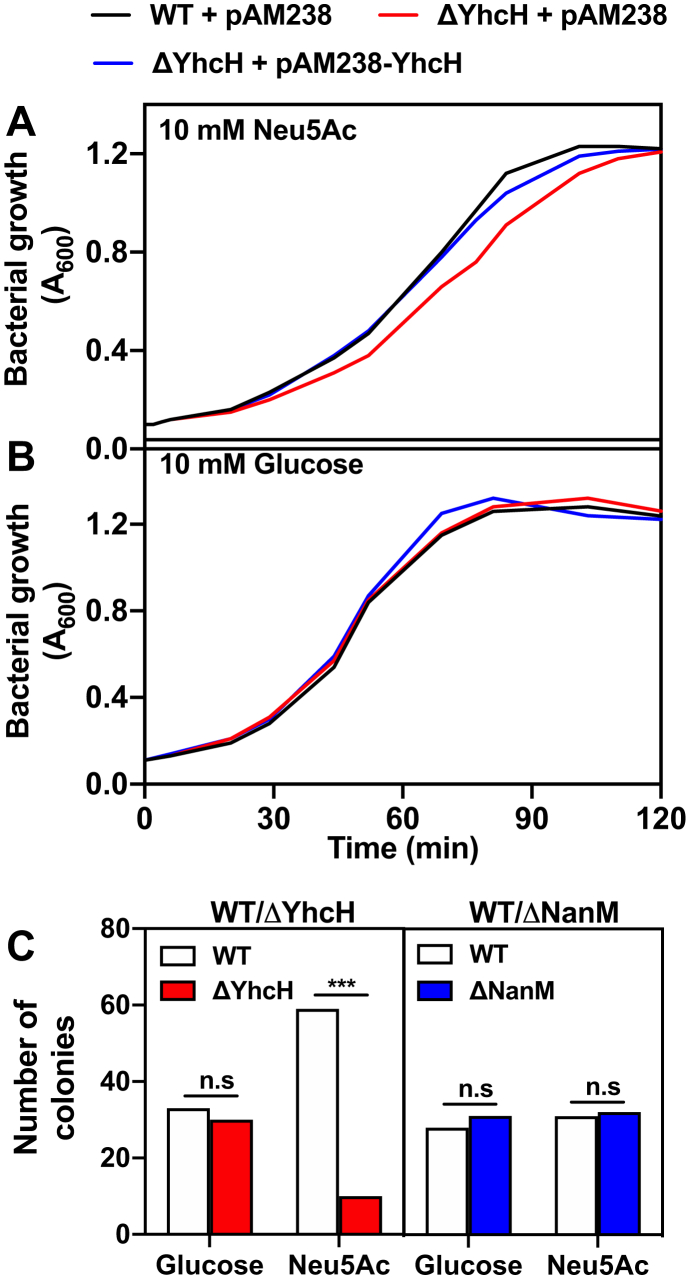
**Effect of YhcH deficiency on growth of *E. coli*.***A* and *B*, wild-type + pAM238, ΔYhcH + pAM238, and ΔYhcH + pAM238-YhcH strains were cultured on M9 medium containing (*A*) 10 mM Neu5Ac or (*B*) 10 mM glucose. See Fig. S4 for replicates. *C*, competitive growth of WT + pAM238 strain *versus* ΔYhcH + pAM238, and WT + pAM238 strain *versus* ΔNanM + pAM238 in M9 medium containing 5 mM Neu5Ac or 5 mM glucose. After six successive full growth cycles, ≈60 colonies were genotyped by PCR for each condition. All growth tests were performed at 20 °C, cultures were shaken at 200 rpm, and the pH of the medium was adjusted to 6.0. n.s, not significant, ∗∗∗*p* < 0.001, by Chi-squared test.

We also tested whether YhcH inactivation affected the growth of *E. coli* in medium containing either glucose or Neu5Ac as carbon source. Growth of the YhcH mutant on Neu5Ac was similar to the wild-type *E. coli* at 37 °C and pH 7, consistent with earlier reports (24) (Data not shown). However, it was slightly but systematically delayed (Fig. 5, *A* and *B* and Fig. S4) at pH 6 and 20 °C, and this was corrected by complementation with YhcH. No growth defect was observed on glucose. We also performed competitive fitness assays in which we mixed equal amounts of wild-type *E. coli* and YhcH (or NanM) deficient bacteria and submitted them to six cycles of exponential growth on medium containing either Neu5Ac or glucose. At the end of the sixth exponential growth period, we plated the cells and genotyped ≈60 colonies by PCR. This revealed that growth on Neu5Ac led to a sixfold decrease in the relative abundance of YhcH-deficient bacteria compared with the wild-type strain (Fig. 5*C*, left panel). This decrease was not observed with NanM-deficient bacteria (Fig. 5*C*, right panel) or when the YhcH deficient bacteria grew on the glucose-containing media (Fig. 5*C*, left panel).

## Discussion

### Preferential action of Neu5Ac aldolase on the open form of substrate

Our results lead to the conclusion that the open form of Neu5Ac form is the best substrate nor Neu5Ac aldolase, even though this form represents less than 0.5% of Neu5Ac in solution (15). This conclusion is supported by the fact that the rate of utilization of both beta and alpha-anomers of Neu5Ac can be increased to a considerable extent by the presence of NanM, a well-known anomerase, whose mechanism of action is highly likely to involve the formation of an open intermediate (19, 20). This acceleration is best observed at acidic pH, a condition that slows down the rate of spontaneous anomerization, and much less at slightly alkaline pH, where the spontaneous anomerization is rapid and is expected to mask the effect of the anomerase.

Our data allow us to rule out the possibility that the anomerases accelerate the action of Neu5Ac aldolase on Neu5Ac by activating the enzyme. A first argument is that it is difficult to envision how NanM, a protein present in the intermembrane space, would interact with NanA, a cytosolic protein. Furthermore, half-maximal activation of NanA activity was observed when NanM was largely substoichiometric (Fig. 1*G*), a behavior difficult to explain through the formation of a protein–protein complex. An additional argument is that even if *E. coli* NanA had a binding site for NanM and/or YhcH, it is highly unlikely that this site would be conserved in human NPL, considering the low degree of amino acid identity (≈31%) between the human and the bacterial aldolases, the considerable phylogenetic distance between the two species and the lack of NanM or YhcH homologs in humans; and yet the bacterial anomerases activated the Neu5Ac aldolase activity of human NPL. Finally, and even more convincingly, NanM and YhcH do not stimulate the action of NanA on 2-keto-3-deoxyxylonate, a compound that is largely present in the open form (22).

Mammalian fructose-1,6-bisphosphate aldolase binds the beta-anomer of fructose-1,6-bisphosphate, and this is followed by opening of the furanose cycle and formation of a Schiff base with a conserved lysine (25, 26). Our data do not completely exclude the possibility that NanA binds and subsequently opens the alpha-anomer as proposed by (27). However, this possibility is extremely unlikely, since in the absence of anomerase, the Neu5Ac aldolase activity does not change with increasing amount of the aldolase, suggesting that formation of the “real” substrate is the limiting element.

Thus, our data indicate that NanA behaves more like the *Pseudomonas putida* 2-keto-3-deoxygluconate 6-phosphate aldolase that was shown to work on the keto form of the substrate, which represents about 9% of the total substrate (28). Of note, in this case, there is presumably no advantage of having an anomerase to facilitate the action of the aldolase, because of the quite high proportion of the keto form.

### A new type of anomerase

Until now the function of the proteins belonging to the DUF386 family has remained unknown (11, 29). We show in this paper that a subset of these proteins act as Neu5Ac anomerases. YhcH is a metalloprotein, unlike NanM, the Neu5Ac anomerase present in the intermembrane space of several Gram-negative bacteria (20), and unlike anomerases acting on galactose (30), L-fucose and L-rhamnose (31, 32). In contrast to previous reports, YhcH in our hands preferentially contains Zn^2+^. The divalent cation plays an important role in catalysis, as indicated by the inhibition observed with metal chelators. Interestingly, we found that divalent cations, in particular Zn^2+^, catalyze the anomerization even in the absence of a protein. This last conclusion is based on the observation that divalent cations (i) promote the action of Neu5Ac aldolases on Neu5Ac; (ii) stimulate the formation of semicarbazone; (iii) and facilitate the conversion of the alpha-form of Neu5Ac to the beta-form when measured by NMR. It should be noted that the concentrations of divalent cations required to observe these effects are nonphysiological. For instance, in *E. coli*, the free Zn^2+^ concentration has been estimated to be 0.1 to 0.2 nM (33). These metal effects are nonetheless interesting to report for future mechanistic studies as well as to avoid assay artifacts.

Anomerization usually proceeds *via* acid-catalyzed hydrolysis of the sugar to the open-chain form, followed by reformation of the cyclic hemiacetal (16, 28). Here, our hypothesis is that the free divalent cation could coordinate the carboxylate and hydroxyl groups on C2 and therefore promotes hemiacetal hydrolysis *via* a Lewis-acid-catalyzed mechanism. This divalent cation might play a similar role in the active site of YhcH, which is therefore one more example of an enzyme whose cofactor has an intrinsic catalytic activity.

### The role of YhcH is to act as an “openase” rather than as an anomerase

Our data indicate that the open form of Neu5Ac is the preferred substrate for the *E. coli* and human aldolases and that YhcH serves to provide this substrate in the cytoplasm. Both YhcH and NanM facilitate the action of the aldolase, suggesting that, in both cases, the open intermediate is not tightly bound to the catalytic site during the catalytic cycle. The structures of both YhcH (11) (Figs. S5 and S6) and NanM (20) are compatible with this: in both cases, the catalytic site is a depression at the surface of the protein and there is no evidence for the presence of a lid that would close the catalytic site during interconversion of the anomers and would thereby prevent leakage of the open intermediate. Of note, YhcH seems to be better at facilitating the utilization of the beta anomer than of the alpha-anomer, for which its anomerase activity is 50-fold lower than that of NanM. This suggests that the role of YhcH activity is to facilitate the utilization of the largely predominant beta anomer and the selective pressure has been exerted on this “openase” activity rather than on the overall anomerase activity.

To the best of our knowledge, our work reveals for the first time that an anomerase’s physiological role can be to provide the open form of a substrate. This is very different from the accepted function of anomerases to provide the adequate anomer for subsequent enzymatic reactions, such as the phosphorylation of C1 of D-galactose (30, 34) and L-fucose (35) by the relevant kinases, or the oxidation of C1 of L-rhamnose by L-rhamnose dehydrogenase (36).

However, other anomerases might serve a similar function to that of YhcH. For example, some L-fucose and L-rhamnose mutarotases (*i.e.*, anomerases) genes are found in or near some bacterial operons encoding also an isomerase that converts the indicated aldoses to ketoses (35, 36). To the best of our knowledge, the anomeric specificity of these isomerases is unknown. As their reaction mechanism logically involves the formation of a linear form of sugar, we speculate that the mutarotases might serve to provide the isomerases with an open substrate. If this would be the case, their presence would be expected to increase several-fold the activity of purified isomerases by constantly replenishing the minor pool of open form of the aldose substrate that would otherwise be rapidly exhausted by the isomerase. Such high stimulation would not be expected if the anomerases served only to equilibrate the anomers, because the anomers are much more abundant species that are not readily exhausted.

### Is it useful to produce an unstable intermediate?

We are postulating that YhcH serves to produce an unstable open form of Neu5Ac that can be used by the aldolase NanA as a substrate. The problem with this model is that the unstable intermediate has to reach the appropriate site where it will be used before decaying to a “useless” form. From the data obtained for the cleavage of Neu5Ac by NanA, at pH 7 and 20 °C (see Fig. 1*C*), we calculate that the rate of formation of the linear form from both the beta and the alpha-anomers is 6.4 nmol/min/ml at 0.2 mM Neu5Ac. Assuming that the open chain form represents ≈0.4% of total Neu5Ac at equilibrium (15), we calculate a rate constant of 0.13 s^−1^ for the spontaneous conversion of the linear form to both alpha and beta-anomers, which corresponds to a half-life of ≈5 s. In the context of diffusion-limited catalysis, the rate of productive encounters of substrate and enzyme has been estimated to be of the order of 10^8^ to 10^10^ M^−1^ s^−1^ (37). Thus if the aldolase is present in the cell at a concentration of 1 μΜ (this is a conservative estimate: a concentration of 30 μM can be calculated based on the purification of the enzyme from *E. coli* grown on Neu5Ac (38)), each molecule of substrate hits an aldolase active site between 100 and 10,000 times per second. This figure amounts to roughly 500 to 50,000 encounters during the calculated half-life of the linear form of Neu5Ac, which is enough for cleavage by the aldolase to occur even if only a small fraction of the encounters leads to catalysis.

### Utility of YhcH

The best evidence for the utility of YhcH is the finding that the YhcH-deficient strain grows slower than the wild-type strain when Neu5Ac is used as a carbon source, but behaves identically on glucose. A further indication is the fact that YhcH is conserved in many genomes of bacteria that can metabolize Neu5Ac, as indicated by the presence of N-acetylneuraminate aldolase, N-acetylmannosamine kinase, and N-acetylmannosamine 6-phosphate epimerase. Of the 21 Neu5Ac metabolizing bacteria listed in Table S2, 18 comprise (at least) one YhcH homolog, which either because of its close proximity to genes involved in Neu5Ac metabolism or because of its high degree of identity with *E. coli* YhcH is very likely to be a Neu5Ac anomerase.

Of note, spontaneous isomerization is highly accelerated by increasing the pH (fivefold increase per pH unit). Accordingly, the effect of anomerase addition was best observed at pH 6, still quite visible at pH 7, and almost unapparent at pH 8 (see Fig. 1, *B*–*D*). The intracellular pH of *E. coli* has been estimated to be between 7.2 and 7.8, depending on the pH of the growth medium (from pH 6 to 9) (39, 40). The acidic (6.0) pH of the growth medium that we used to study the effect on growth of YhcH deficiency was presumably favorable to observe this effect. It is likely that little or no effect of YhcH deficiency would be observed at a more alkaline pH.

The proposed role of YhcH contrasts with that of NanM in *E. coli*. The transport of Neu5Ac by NanT is suggested to be specific for the beta-anomer, and NanM would therefore make the alpha-form available for *E. coli* by accelerating its conversion to the beta-form (20). This is particularly important if one considers that sialidases produce the alpha-form of N-acetylneuraminate, which is an inhibitor of sialidases. The presence of NanM in the intermembrane space may, therefore, enhance the availability of Neu5Ac by decreasing sialidase inhibition.

No homologue of YhcH is found in vertebrates. Kelch proteins are homologues of NanM, but they do not comprise the region that contains the residues involved in catalysis (20), and are therefore unlikely to catalyze the anomerization of Neu5Ac. This suggests that vertebrates, which can metabolize Neu5Ac with the appropriate aldolase, either have no Neu5Ac anomerase or have a form that is totally different from YhcH and NanM. Considering the low rate of Neu5Ac metabolism in vertebrates, the lack of an anomerase is likely not a problem.

## Conclusion

In contrast to what had been previously suggested, our data show that Neu5Ac aldolase preferentially uses the open form of its substrate and that YhcH provides this form of substrate in *E. coli* and many other bacteria, thereby conveying a competitive advantage for the growth on Neu5Ac. This is the first time that an anomerase is shown to provide the unstable open form to another enzyme, but other examples likely remain to be discovered. Beyond this, the identification of the function of YhcH represents the first functional identification of a DUF386 family member, thereby opening the door to future investigations into the function of other members. More generally, our study points to the importance of searching for as yet unknown enzymes that accelerate spontaneous reactions and might play a critical role under some specific conditions.

## Experimental procedures

### Bacterial strains

The clones encoding YjhC, the Neu5Ac aldolase NanA, and the Neu5Ac mutarotase NanM (20) were from the ASKA library, which comprises a complete set of ORF clones of *E. coli* K-12 strain (21). ORFs are cloned in the pCA24N vector in which a His6-tag is located at the N-terminal side of the cloning multiple site (21). All primers used in this work were from Integrated DNA technologies.

Strain *E. coli* K12 *yhcH::kan* and *nanM::kan* were obtained from the Keio collection (41). The kanamycin resistance cassette was excised using the pCP20 plasmid-mediated protocol (42) to generate *ΔyhcH* and *ΔnanM* mutants. pAM238 vector was used to rescue *yhcH* in the *ΔyhcH* null mutant. DNA sequences were verified by DNA sequencing performed by Genewiz.

### Preparation of YhcH and human NPL in pET22b(+) vector

The *yhcH* and human *NPL* genes were PCR-amplified using the DNA polymerase Phusion (Thermo Scientific) from *E. coli* genomic DNA and human liver cDNA, respectively. The primers used for *yhcH* were: forward primer 5’-GGGTTGCATATGATGATGGGTGAAGTACAG-3’ (NdeI restriction site is underlined) and reverse primer 5’-CCAACCCTCGAGAGCCATTAAATCAGCCTTAACC-3′ (XhoI). For human *NPL*: forward is 5’-GAGACATATGATGGCCTTCCCAAAGAAGAAA-3’ (NdeI) and 5’-AAATGCGGCCGCGCTACCAGCTTCCAAGTTTC-3’ (NotI) for the reverse one. The resulting PCR products were purified and digested with their respective restriction enzymes (all from Thermo Scientific). The purified and digested PCR products were ligated with predigested pET22b(+) vector and transformed into chemically competent *E. coli* XL1 Blue cells. Selected clones were grown to prepare minipreps and DNA sequences were confirmed by DNA sequencing. The recombinant vectors were then transformed into chemically competent *E. coli* BL21 (DE3) cells more adapted for protein expression.

### Expression and purification of recombinant enzymes

NanA, NanM, and YjhC were expressed as previously described (6). For YhcH and human NPL expression, *E. coli* BL21 recombinant strains were cultured in LB medium (10 g/l tryptone, 5 g/l yeast extract and 10 g/l NaCl) supplemented with ampicillin at 200 μg/ml. ZnCl_2_ (30 μM) was added to the culture medium to obtain Zn-saturated YhcH, as used in the enzymatic assays. Expression of the enzymes was achieved by induction with 0.1 mM of isopropyl-1-thio-ß-D-galactopyranoside (IPTG) when OD_600nm_ reached 0.4 to 0.6. This was followed by incubation at 37 °C for 20 h with shaking at 200 rpm.

The cells were harvested by centrifugation at 6000*g* for 20 min. His6-tagged protein purifications were performed using the AKTA purifier 900 series (GE Healthcare) by using a Ni^2+^-resin column (HisTrap-HP 1 ml, GE Healthcare) as described previously (5).

Purity of the recombinant proteins was estimated by SDS-PAGE and Coomassie Blue staining. SDS-PAGE was performed in commercial NuPageTM 10% BisTris-glycine gels (Invitrogen). PageRuler plus Prestained Protein Ladder 10 to 180 kDa (Thermo Scientific) were used as molecular weight standards. Gels were stained with Coomassie Blue R-250.

Protein concentration in the purified preparations was estimated by measuring A_280_ and computing the concentration from the expected extinction coefficient on the basis of the amino acid composition (Protparam tool, at https://web.expasy.org/protparam/).

### Measurement of Neu5Ac aldolase activity

The measurement was performed spectrophotometrically at 340 nm with a Beckman Coulter DU-800 series machine. The reaction mixture contained 50 mM MES, pH 6.0, 10 mM KCl, 0.5 mM DTT, 1 mM MgCl_2_, 0.5 mg/ml bovine serum albumin, and 150 μM NADH at 20 °C. HEPES buffer was used for assays at pH 7.0 and 8.0. The mixture was supplemented with 200 μM Neu5Ac. The coupling enzyme lactate dehydrogenase (Roche) was added to the mixture (5.0 μM). The Neu5Ac mutarotase NanM was added in some assays at 0.5 μM. The reaction was started by adding the aldolase NanA at 1.5, 3, or 6 μM in a final volume of 600 μl.

### Enzymatic measurement of the activity of NanM or YhcH

The reaction mixture contained 50 mM MES buffer, pH 6.0, 10 mM KCl, 1 mM MgCl_2_, 0.5 mg/ml bovine serum albumin, and 150 μM NADH. The mixture was supplemented with 120 μM Neu5Ac in a final volume of 600 μl. For some assays Neu5Ac was replaced by 2-keto-3-deoxy-D-glycero-D-galacto-nonoate (KDN) or 2-keto-3-deoxyxylonate (both from Sigma-Aldrich). LDH (5.0 μM) was added to the mixture as coupling enzyme. YjhC (3 μM (6)) or *Clostridium perfringens* sialidase A (0.6 U/ml, Sigma-Aldrich) was also added when the substrates were 2,7-anhydro-Neu5Ac (2,7-AN) or 3’-siallylactose, respectively. NAD^+^ was also added at 50 μM when YjhC was used (6). Several concentrations of NanM or YhcH were tested. 2 μM NanA (or 6.0 μM human NPL) was used to start the reaction. Measurements were performed spectrophotometrically at 340 nm at 20 °C.

### Stimulation of the formation of a semicarbazone from Neu5Ac or 2-keto-3-deoxyxylonate by NanM and YhcH

The reaction mixture contained 100 mM phosphate potassium, pH 6.0, and 10 mM semicarbazide (Sigma-Aldrich). The assay was started by adding 120 μM Neu5Ac or 2-keto-3-deoxyxylonate. The anomerases YhcH or NanM were added at 5 μM and 0.8 μM, respectively. The formation of semicarbazone was monitored spectrophotometrically at 249 nm and 20 °C (23).

### Metal analysis by ICP-MS

For the determination of the metal content of YhcH and other proteins, the purified protein preparation was freed from unbound metals by gel filtration on a NAP-5 column (GE HealthCare) equilibrated with metal-free water (Millipore) according to the manufacturer’s recommendations. The metal content was quantified by means of inductively coupled plasma–mass spectrometry (ICP-MS) on an Agilent 7500cx instrument. Briefly, protein preparations were diluted tenfold with a 1-butanol (2%w/v), EDTA (0.05%w/v), Triton X-100 (0.05%w/v), NH_4_OH (1%w/v) solution containing Sc, Ge, Rh, and Ir as internal standards.

### Effect of inhibitors on YhcH activity

The impact of some potential inhibitors on YhcH activity was assessed. The tested concentrations were from 100 μM to 1 mM for 1,10-phenanthroline and from 1 to 8 mM for Bestatin, DTT, EDTA, or EGTA (all from Sigma Aldrich). The reaction mixture contained 50 mM MES, pH 6.0, 10 mM KCl, 1 mM MgCl_2_, 0.5 mg/ml bovine serum albumin, and 150 μM NADH. The mixture was supplemented with 200 μM Neu5Ac and the final volume was 600 μl. NanA (2.0 μM) and LDH (5.0 μM) were added to the mixture as coupling enzymes. The mixture contained 0.8 μM YhcH or 0.08 μM NanM. The measurements were performed spectrophotometrically at 340 nm and 20 °C.

### Effect of divalent cations on the stimulation of Neu5Ac aldolase activity

The measurement was performed spectrophotometrically at 340 nm. The reaction mixture contained 50 mM MES, pH 6.0, 10 mM KCl, 0.5 mM DTT, 0.5 mg/ml bovine serum albumin, and 150 μM NADH at 20 °C. The mixture was supplemented with 120 μM Neu5Ac. LDH (5.0 μM) was added as a coupling enzyme. CaCl_2,_ MnCl_2_, MgCl_2_, NiCl_2_, CoCl_2_, and CdCl_2_ were tested up to 10 mM. Metal solutions were titrated by ICP-MS. The reaction was started by adding 2 μM NanA in a final volume of 600 μl.

### Effect of divalent cations on the stimulation of the formation of a semicarbazone from Neu5Ac

The reaction mixture contained 100 mM potassium phosphate, pH 6.0, and 10 mM semicarbazide. The assay was performed at 20 °C. The reaction was started by adding Neu5Ac at 120 μM. MnCl_2_, MgCl_2_, and CaCl_2_ were tested up to 15 mM. ZnCl_2_, NiCl_2_, and CoCl_2_ were tested up to 10 mM. The formation of semicarbazone was monitored spectrophotometrically at 249 nm (23).

### Growth of *E. coli* mutants on Neu5Ac

M9 minimal medium (Sigma Aldrich; 33.9 g/l Na_2_HPO_4,_ 15 g/l KH_2_PO_4,_ 5 g/l NH_4_Cl, 2.5 g/l NaCl, 223 mg/l MgCl_2_) supplemented with 100 μM CaCl_2,_ 1 mM MgSO_4_, and 100 μg/ml kanamycin, was used to assess the ability of *E. coli* ΔYhcH + pAM238 and ΔYhcH + pAM238-YhcH strains to grow on Neu5Ac. The medium was supplemented with 10 mM glucose or Neu5Ac as energetic substrate. After overnight growth, 30 ml of the Neu5Ac- (or glucose-) containing M9 was inoculated at the OD of 0.1. Growth tests were performed at 20 °C and 200 rpm for 120 h.

### Competitive growth of wild-type and YhcH deletion strain on Neu5Ac

Growth tests were performed in M9 minimal medium containing 100 μM CaCl_2,_ 1 mM MgSO_4_, and 100 μg/ml kanamycin. The medium was supplemented with 5 mM glucose or Neu5Ac as energetic substrate. Overnight cultures of WT, ΔyhcH, and ΔNanM strains (all containing the empty plasmid pAM238) were centrifuged and pellets were resuspended in M9 medium. Cocultures of WT and ΔYhcH and those of WT and ΔNanM were initiated by addition to the culture medium (10 ml) of an amount of bacterial suspension corresponding to 0.1 OD for each strain. After 24 h of growth at 20 °C, 2 ml of the culture was used to inoculate an 8 ml M9 fresh culture medium containing 5 mM of Neu5Ac or glucose. Six growth cycles were realized successively. At the end of the sixth culture period, serial dilutions were plated on LB agar containing 100 μg/ml kanamycin. The isolated colonies were genotyped by PCR by amplifying the region of *yhcH* or *nanM* genes. For *yhcH* screening, the forward primer was 5’-AGGAGAGAATTCTGATTTACTGGCGGCGCATT-3’ and 5’- GGGAGACTGCAGTTAAGCCATTAAATCAGCCTTAA-3’ for the reverse one. For *nanM* screening the forward primer was 5’-AGGAGAGAATTCGCAGAGATAATTTACGGAAAAC-3’ and the reverse primer was 5’-AGGAGAGTCGACTTAGTTTTGTACTGTGACTTT-3’.

### NMR analysis

All experiments were performed on a Bruker Ascend Avance III 600 MHz system equipped with a broadband cryoprobe (Bruker). Experiments were conducted at pH 5.5 or 6.0 at 10 °C in 10% D_2_O containing 50 mM sodium deuterated acetate buffer, 10 mM NaCl, and 100 μM 2,2,3,3-^2^H_4_ (trimethylsilyl)propionic acid sodium salt (TMSP) as a chemical shift reference (0 ppm). Water signal suppression was achieved using an excitation-sculpting scheme. Thirty-two scans were collected for each spectrum to yield a 15K-point free induction decay.

To assess the anomerase activity of YhcH, 1 mM 2,3-EN was used as starting substrate in the presence of 20 μM NAD^+^ and 1.5 μM of the hydratase YjhC, which converts 2,3-EN to a mixture of 2,7-AN and α-Neu5Ac (6). Anomerases YhcH and NanM were tested at concentrations of 15 μM and 0.5 μM, respectively. We monitored the time course of appearance of shifts specific to H3 axial for both α- and β-Neu5Ac.

To study the anomerization of Neu5Ac catalyzed by divalent cations, 0.6 U/ml of *C. perfringens* sialidase A (Sigma Aldrich) was used to hydrolyze 1 mM 3’-sialyllactose to lactose and α-Neu5Ac. CoCl_2_, NiCl_2_, CaCl_2_, MgCl_2_ were tested at 5 mM and ZnCl_2_ was tested at 1 mM. We monitored the time-dependent evolution of peaks corresponding to H3 axial of the alpha-anomer and H3 equatorial of beta-anomer of Neu5Ac.

## Data availability

All data are shared in the main article or in the supporting information.

## Supporting information

This article contains supporting information.

## Conflict of interest

The authors declare that they have no conflicts of interest with the contents of this article.
