# Supplementary material for: The metalloprotein YhcH is an anomerase providing N-acetylneuraminate aldolase with the open form of its substrate
Source: J Biol Chem. 2021 Apr 23;296:100699. doi: 10.1016/j.jbc.2021.100699 (PMC8141875; doi:10.1016/j.jbc.2021.100699)
Supplement: Figures S1–S6 and Tables S1–S2 [file mmc1.pdf]

The metalloprotein YhcH is an anomerase providing N-acetylneuraminate aldolase with the open form of its substrate

Takfarinas Kentache[1], Leopold Thabault[2], Gladys Deumer[3], Vincent Haufroid[3], Raphaël Frédérick[2], Carole L. Linster[4], Alessio Peracchi[5], Maria Veiga-da-Cunha[1], Guido T. Bommer[1,6], and Emile Van Schaftingen[1*].

*Emile Van Schaftingen
Email: emile.vanschaftingen@uclouvain.be

List of supporting informations:

Figures S1 to S6
Tables S1 to S2

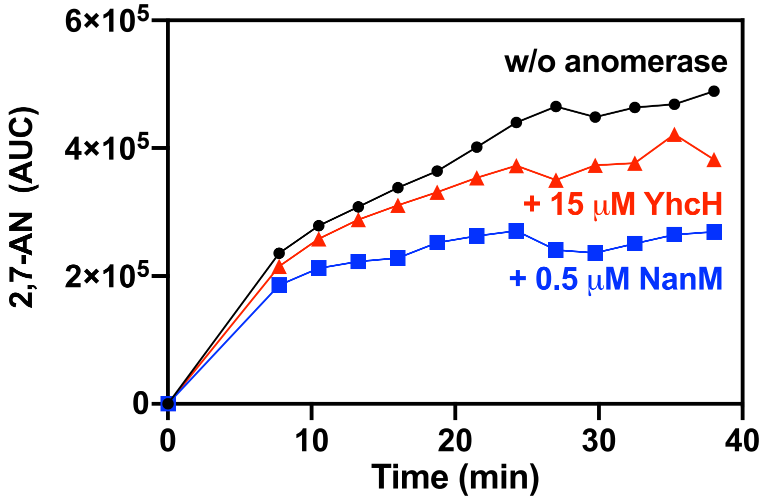

Fig. S1. ^{1}H-NMR data showing the evolution of 2,7-AN in the experiment shown in Fig. 2D-G. The amount of 2,7-AN was calculated from the shift of N5-H in the 8 ppm region.

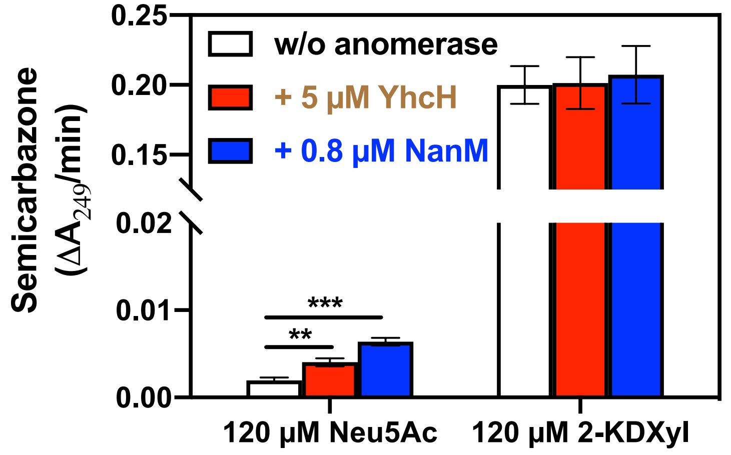

Fig. S2. Stimulation of the formation of a semicarbazone from Neu5Ac or 2-keto-3-deoxyxylonate by NanM and YhcH. The assays were performed at 20°C in 100 mM potassium phosphate, pH 6.0, in the presence of 10 mM semicarbazide, 120 µM Neu5Ac or 2-keto-3-deoxyxylonate (2-KDXyl), and 5 µM YhcH or 0.8 µM NanM. The formed semicarbazone was monitored spectrophotometrically at 249 nm. Results shown are means ± SD (n=3). **: $p < 0.005$, ***: $p < 0.001$ by t-test.

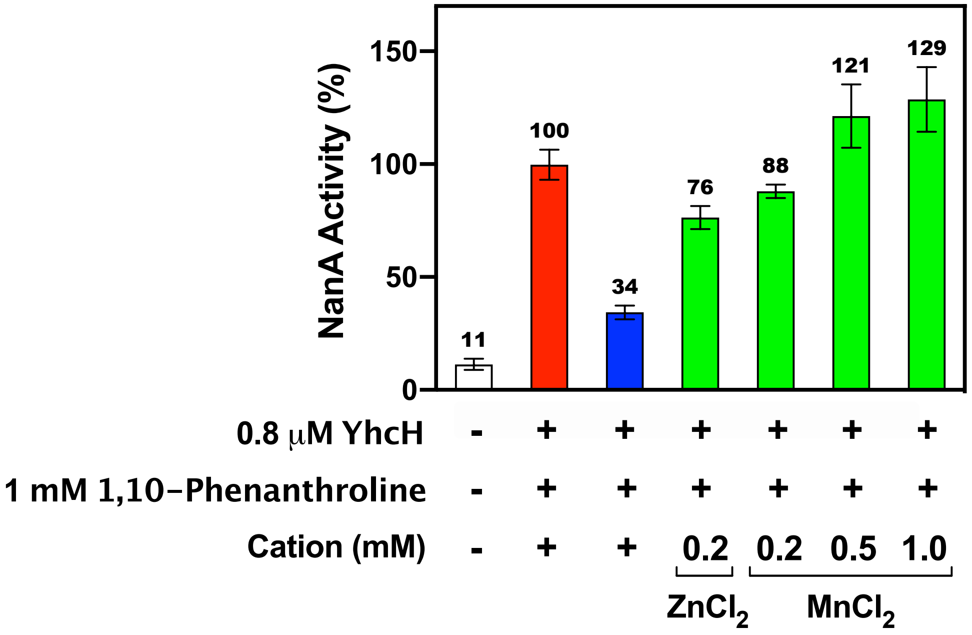

Fig. S3. Recovery of the 1,10-phenanthroline inhibited YhcH activity with Zn^{2+} or Mn^{2+}. The reaction mixture contained 50 mM MES, pH 6.0, 120 μM Neu5Ac, 2 μM NanA, 0.8 μM YhcH, 150 μM NADH, 1 mM 1,10-phenanthroline and the indicated concentrations of $ZnCl_2$ or $MnCl_2$. Assays were performed at 20°C. Results shown are means ± SD (n=3).

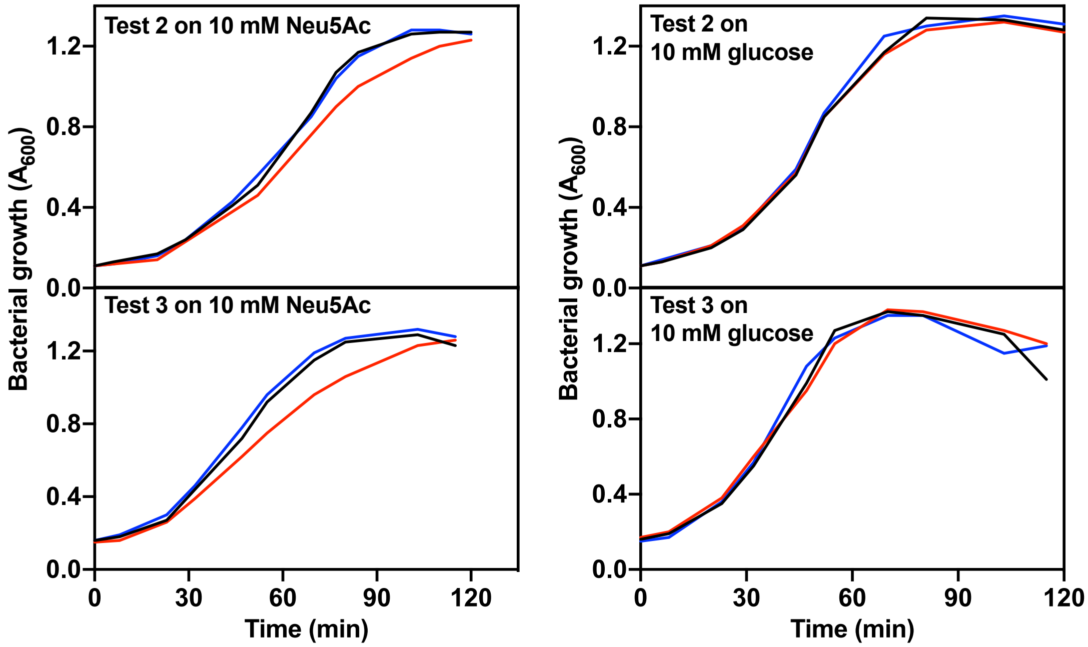

Fig. S4. Effect of YhcH deficiency on the growth of *E. coli.* The figure shows the results of two additional independent experiments performed under the same conditions as in Fig. 5A and 5B.

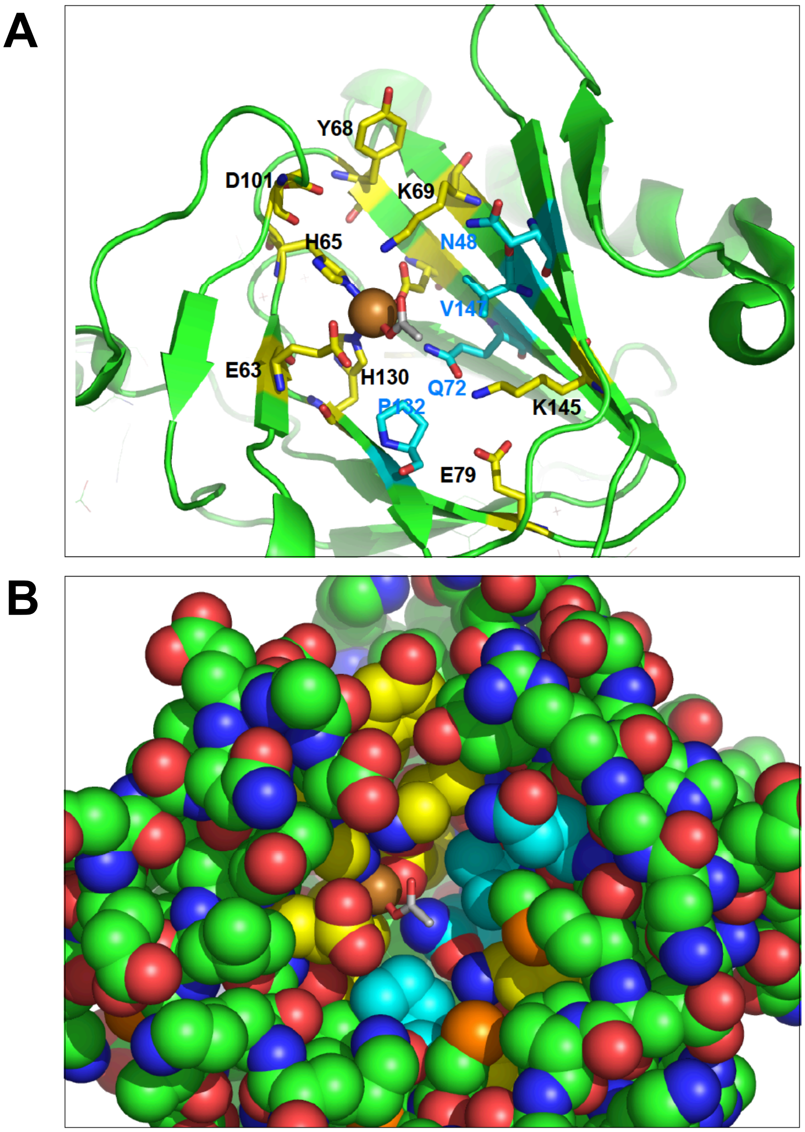

Fig. S5. Structure of *H. influenzae* 10810 YhcH showing conservation of the residues in the putative catalytic site. See ref (1) for the structure of *H. influenzae* YhcH. **A**. Cartoon model showing the strictly conserved residues in the alignment presented in Fig. S6 as yellow sticks and the highly conserved residues of the putative catalytic site in cyan. The copper is shown as a brown sphere and the bound acetate in grey sticks. **B**. Space filling representation of the same region showing that the putative catalytic site is open. Drawn with PyMol Molecular Graphics System 2.0 (2).

```
R.gnavus AGR2154-(p2363)                  MIFDSIQNKENYKEDRKIYQALCYLAEIKRWEEAFPD-TVIQKGVIVANPVSFV-SKEEKECMYEAHQKYIDVHYIIEGV 78
E.coli K12-(p3165)                        MMMGEVQSL-PSAGLHPALQDALTLALAARPQEKAPGRYELQGDNIFMNVMTFN-TQSPVEKKAELHEQYIDIQLLLNGE 79
Y.pestis 2501-(p1649)                     MISGSLAQPAFFSGLPERLQWVLAYLADMPLAQLSCGRHEIDGELMFMNVMAFD-TQPAESKKAELHRTYADVQLLISGi 79
H.somnus 129PT-(p735)                     MIFGDLTRDDFKRNLPQVMADICNRLNTLDLENLTTGRHDLTD-QIYMNVMEST-TVASEDKKAELHHQYIDIQVLISGA 78
H.influenzae 10810-(p310)                 MIISSLTNPNFKVGLPKVIAEVCDYLNTLDLNALENGRHDIND-QIYMNVMEPE-TAEPSSKKAELHHEYLDVQVLIRGT 78
G.anatis UMN179-(p2241)                   MFIGDLTKAEYKLGLPKVIVEALDHLQTLDLAGLELGKHNITD-QIYMNVMEFD-TTDAGSKKAEIHRQYLDIQVLISGK 78
P.multicoda 2000-(p425)                   MFIGDLTRNDFKLGLPPVIAEVCDYLNTLDLAALETGRHDITE-QIYMNVMAFD-TAAAESKQAELHRQYIDVQVLISGQ 78
A.actinomycetemcomitans D11S-1-(p1947)    MIIGDLTRADVKLGLPPVIAQICDYLNGLDLTALTNGRHNITD-QIYMNVMEFD-TAPADSKQAELHHKYLDVQVLISGA 78
S.fluaggei ATCC43531-(p1387)               MIYGSIHHEDTYSFLPPRIRQALDFARTHDLGALPTGRCEIAGDDVYVNIAHYT-TGERSEKIWEAHRAYIDIHVLAEGT 79
C.perfringens ATCC13224-(p167)            MIFGNLINLESYDYLPKLVKEALLHAKNNNVIEFEPGTHEIKGEELFFNRVNYT-TKNKEDRFWEGHKKYIDVHVVFSGR 79
S.pneumonaie TIGR4-(p1232)                MIISEQSDFKRYASVNKYFSKVCDFLENTNLTDLVDGKIDIDGENVFANCMTY-LADGVPGDIFETHKKYLDIHIVVENT 79
S.pneumonaie TIGR4-(p1577)                MIITKISRLGTYVGVNPHFATLIDFLEKTGLENLTEGSIAIDGNRLFGNCFTY-LADGQAGAFFETHQKYLDIHLVLENE 79
S.pneumonaie TIGR4-(p1566)                MIFDDLKNITFYKGIHPNLDKAIDYLYQHRKDSFELGKYDIDGDKVFLVVQENVLNQA-ENDQFEYHKNYADLHLLVEGH 79
S.pyogenes ATCC10782-(p1566)               MIYDRLKNISRYRGIHPHLDAAITFLQTTDLRQLAEGKYPILGEKVFAVIQRNQLSKA-DNSLLEYHKRYADCHLLLAGN 79
M.capricolum ATCC27343-(p413)             MIYDKISNLNRYLNIHKNIDLAIKFIQEHDLKSLKDGINVINDKLFYNKFTTKTL--DINDAIFESHDKYLDLHIIISGD 78
F.gonidiaformans ATCC25563-(p325)         MIYDKIENIGRYLGISNYLDQAIRYIMTGNYQKAEYGRNVVAGEDIYYNCPEGAMAKNVEGMDYEYHRTYIDIHIPLKGK 80
F.nucleatum animalis 11_3_2-(p177)        MIYAELKNIKTYKGINKNLDKAIDFIAEKKYMNASFGKNIIEGDTIYFNYPEKPMTRENIDLDLEYHKKYIDIHIVLEGE 80
                                          *:                                  .   :    .                  * *  : * :    .

R.gnavus AGR2154-(p2363)                  ERIATADVSS-LQIQTPFSEEKDIGFYQGDE-SGGYLLKAGEFMVCFPSDAHKVAMMDKS-PEIVKKIVVKIKV----- 149
E.coli K12-(p3165)                        ERILFGMAGT-ARQCEEFHHEDDYQLCSTIDNEQAIILKPGMFAVFMPGEPHKPGCVVGEPG-EIKKVVVKVKADLMA- 154
Y.pestis 2501-(p1649)                     EGIEYSTLTPTE-HLEPYHPDDDYQLIADIPDKSQLRMLPGMFAVFLPGEPHKPGCQIAGSG-AIKKVVVKVHYSLLNS 156
H.somnus 129PT-(p735)                     ETIEYGVEYPNLADCTAYNEQDDYQLIDDIPHKSAVTLRPKMFAIFFPYEPHKPCCNVHGQVTHLKKLVVKIPISLID- 156
H.influenzae 10810-(p310)                 ENIEVGATYPNLSKYEDYNEADDYQLCADIDDKFTVTMKPKMFAVFYPYEPHKPCCVVNGKTEKIKKLVVKVPVKLI-- 155
G.anatis UMN179-(p2241)                   EAIQCSPVYPDLSLYTEYDEKDDYQLTPDIDAKSEIVLRPKMFAIFLPYEPHKPGCNVEGKVQHLKKLVVKIPVTLL-- 155
P.multicoda 2000-(p425)                   ENIEYSVAYPTLANYTEYDEKDDYQLTPEIEYKSTLTLKPKMFVVFLPYEPHKPGCNVDNQVVSLKKLVVKVPVELL-- 155
A.actinomycetemcomitans D11S-1-(p1947)    ESIDYSVTHPDLNQYTDYNDADDYQLCPIIEQQNTLTLRPKMFVVFYPYEPHRPGCNVNGQTVKLKKLVVKIPVALIK- 156
S.fluaggei ATCC43531-(p1387)               EKIDVSLI-E-RMETHPYEADKDYVPADGAV-TQTTVMRPGDFLVCFPEDVHRSGVKVDA-PAPLKKGIFKVRV----- 149
C.perfringens ATCC13224-(p167)            ERIDLNFK-E-NLEFKEFVEKDDFLSFDGKE-NSTVVLEEGDFLICYPEDIHMTCLKADNEPSDVKKAIYKVIL----- 150
S.pneumonaie TIGR4-(p1232)                EKMAVTSPVR-AQSRVPFSEEKDIAFYDSKD-YQIVELLPGNMLVTFEEDLHQPKIHCND--ETVKKLVIKVLNEEK-- 152
S.pneumonaie TIGR4-(p1577)                EAMAVTSPEN-VSVTQEYDEEKDIELYTGKV-EQLVHLRAGECLITFPEDLHQPKVRIND--EPVKKVVFKVAIS---- 150
S.pneumonaie TIGR4-(p1566)                EYSSYGSR-I-KDEAVAFDEASDIGFVHCHE-HYPLLLGYHNFAIFFPGEPHQPNGYAGME-EKVRKYLFKILID---- 150
S.pyogenes ATCC10782-(p1566)               ECIRYGIG-N-QAEVVPFEQEADIGFVTCDR-TYDLDLVDDSFAYFFPNEPHQPNNFNQAG-EAVTKCLIKVLMAD--- 151
M.capricolum ATCC27343-(p413)             EYIGHEFVSD-LKEQVLYNQKDDVYLYKTKT-TKTIYQNDQSFSLFFSTDAHSPKIKANF--NQITKIVFKILYD---- 149
F.gonidiaformans ATCC25563-(p325)         ENIAFFEMKQ-GKEVKAYEEENDYGLYQGIA-EGKLCIKEGEFLMLFPEEVHLALMKVEEEATPIEKVIFKVRAK---- 153
F.nucleatum animalis 11_3_2-(p177)        ESIAYTPFED-CIETKSYNVEEDYGLVKGKA-QAELILNGKNFLLFFPEEPHLALLKVNTP-KEIKKIIFKVEL----- 151
                                          *                :    *                          : *            : * : *:
```

Fig. S6. Multiple alignment of several YhcH's. Alignment was performed with Clustal Omega (www.ebi.ac.uk) (3) using sequences corresponding to the peg numbers indicated in Table S2. Strictly conserved residues are highlighted in yellow and semi-conserved residues present in the putative catalytic site are highlighted in cyan. Blue boxes indicate residues involved in the metal coordination shell in YhcH of *H. influenzae* 10810 (1).

Table S1. Metal content of different preparations of YhcH and of other proteins. The growth condition is indicated. Values were expressed in mol of metal/mol of protein. Abbreviation: ec: *E. coli,* hi: *H. influenzae*

Protein	Growth condition	Mn^{2+}	Ni^{2+}	Cu^{2+}	Zn^{2+}
ecYhcH	LB	0.021	0.061	0.051	0.41
	LB + 80 μM $CuCl_2$	0.015	0.044	0.055	0.467
	LB + 30 μM $ZnCl_2$	0.021	0.039	0.043	1.133
	LB + 60 μM $MnCl_2$	0.873	0.052	0.028	0.308
	M9	0.004	0.021	0.022	0.344
	M9 + 60 μM $MnCl_2$	0.254	0.026	0.009	0.394
	M9 + 30 μM $ZnCl_2$ + 60 μM $MnCl_2$	0.036	0.043	0.008	1.078
	M9 + 30 μM $ZnCl_2$	0.039	0.042	0.011	0.887
ecNanM	M9	0.011	0.092	0.055	0.044
hiYhcH	LB	0.034	0.019	0.014	0.881
ecNanA	LB	0.001	0.026	0.013	0.016

Table S2. Occurrence of YhcH and other enzymes of Neu5Ac metabolism in bacterial genomes. The table was built using data obtained from www.theseed.org (4). Blast searches were performed with the proteins indicated in the top line and the percentage identity and scores are indicated. The identified orf's are indicated with their peg number (abbreviated with a 'p' followed by a number, e.g. p3165) to indicate their relative position in the genome. Genes that are close to other genes involved in sialic acid metabolism are highlighted in cyan, yellow or green. Note that NanM from *Clostridium perfringens,* some other Gram-positive bacteria and *Fusobacterium* are circularly permuted compared to *E. coli* NanM, but that they keep the catalytic residues. Analysis of the sequences by SignalP 5.0 (5) indicates the presence of a signal peptide (SP), indicating that these enzymes are not able to replace YhcH in the cytosol.

The table indicates also that there is only a low degree of amino acid identity between YhcH's from Gram-positive and Gram-negative bacteria. This is reminiscent of the low degree of identity between their N-acetylmannosamine kinases.

Most bacteria capable of metabolizing N-acetylneuraminate possess at least one YhcH homolog that is likely play the role of a Neu5Ac anomerase (see Results). The main exceptions are *Staphylococcus aureus* and *Lactobacillus plantarum*, which do not have any homolog of YhcH (or NanM). In the case of *Vibrio cholerae*, the YhcH homolog shows only a low degree of identity with *E. coli* YhcH, is not present in the Neu5Ac operon and is not conserved in several *Vibrio*'s that are able to metabolize Neu5Ac. It is therefore unlikely to be involved in Neu5Ac metabolism. Of note, *V. cholerae* and many other *Vibrio*'s have two NanM homologs. As previously pointed out (6), one is shorter at its N-terminal extremity than the other one and is missing the predicted signal peptide usually found in NanM homologs. We speculate that the shorter protein is a cytosolic form that replaces YhcH and helps the aldolase to metabolize Neu5Ac.

**Species**	**Gram**	**YhcH** ***Escherichia coli* K12**	**NanM** ***Escherichia coli* K12** **NanM** ***C. perfringens***	**Sialidase NeuA /NeuB** ***Streptococcus agalactiae* 2603VR**	**NanA Aldolase** ***Escherichia coli* K12**	**NanE Epimerase** ***Escherichia coli* K12**	**NanK Kinase** ***Escherichia coli* K12**
Escherichia coli K12 **Enterobacteriales**	**Gram-**	p3165 100 % Sc324	P4220 100% Sc747 SP present	None	p3169 100% Sc613	p3167 100% Sc464	p3166 100% Sc578
Haemophilus influenzae 10810 **Pasteurellales**	**Gram-**	p310 46 % Sc105 isolated	p210 43% sc320 SP present	None	p200 37% Sc200	p203 61% Sc268	p202 49% Sc271
Haemophilus parainfluenzae T3T1 **Pasteurellales**	**Gram-**	p187 45% Sc95 isolated	None	None	p65 38% Sc209	p68 63% Sc279	p67 53% Sc284
Haemophilus somnus 129PT **Pasteurellales**	**Gram-**	p732 46% Sc108	None	None	p730 37% Sc209	p734 62% Sc279	p733 49% Sc258
Pasteurella multocida 2000 **Pasteurellales**	**Gram-**	p425 42% Sc110	p418 38% Sc233 SP present	p1703 39% Sc307 p959 25% Sc37.7	p427 37% Sc208	p423 62% Sc268	p424 51% Sc263
Aggregatibacter actionmy-cetemcomitans D11S-1 **Pasteurellales**	**Gram-**	p1947 42% Sc104 Isolated	p1484 43% Sc304 SP present	None	p1478 37% Sc204	p1481 59% Sc275	p1480 50% Sc272
Actinobacillus pleuropneumonie serovar 1 str 4074, 2e sequence, **Pasteurellales**	**Gram-**	p771 49% Sc102	None	None	p1884 36% Sc197	p1882 60% Sc265	p1883 51% Sc275

Species	Gram	YhcH *Escherichia coli* K12	NanM *Escherichia coli* K12 NanM *C. perfringens*	Sialidase NeuA /NeuB *Streptococcus agalactiae* 2603VR	NanA Aldolase *Escherichia coli* K12	NanE Epimerase *Escherichia coli* K12	NanK Kinase *Escherichia coli* K12
Vibrio cholerae 12129(1) **Vibrionales**	**Gram-**	None	p2304<br>35% sc220<br>SP present<br>p2305<br>30% sc177<br>SP absent	p2295<br>28% Sc47	p2302<br>29% Sc 105	p2298<br>57% Sc242	p2297<br>41% Sc202
Yersinia pestis 2501 **Enterobacteriales**	**Gram-**	p1649<br>47 % Sc 28	p621<br>39% Sc269<br>SP present	None	p1654<br>28% Sc269	p1653<br>61% Sc198	p1650<br>50% sc269
Gallibacterium anatis UMN179 **Pasteurellales**	**Gram-**	p2241<br>45% Sc60	p1662<br>42% Sc307<br>SP present	None	p2243<br>36% Sc213	p2239<br>59% Sc265	p2240<br>51% Sc276
Fusobacterium nucleatum animalis 11_3_2 **Fusobacteriales**	**Gram-**	p177<br>27% Sc60 Yhch *E. coli*<br>35% Sc90 YhcH S. p 1566	p1340<br>26% Sc91<br>50% Sc333<br>Circularly permuted<br>SP present	None	p1345<br>34%	p1346<br>38%	p1344<br>30%
Fusobacterium gonidiaformans ATCC25563 **Fusobacteriales**	**Gram-**	p325<br>30% Sc54	p318<br>28% sc86<br>47 % Sc328<br>Circularly permuted<br>SP present	None	p323<br>36% Sc209	p324<br>40% sc177	p322<br>34% Sc55
Selenomonas fluaggei ATCC43531 **Selenomonadales**	**Gram-**	p1387<br>30% sc65	p1389<br>31% Sc65<br>44 %<br>Circularly permuted<br>SP present	None	p1385<br>36% Sc203	p1384<br>43% Sc164	p1388<br>27% Sc61
Streptococcus pneumoniae Pneumoniae D39 **Lactobacillales**	**Gram+**	p1615<br>29% Sc62.8<br>p1626<br>29% Sc48.5	p1274<br>22% Sc53<br>27 % Sc109<br>Circularly permuted<br>SP absent	p1628<br>NeuA 100% sc2107<br>p1622<br>NeuB 100% sc1431	p1610<br>30% Sc112<br>p1265<br>31% Sc112	p1620<br>38% Sc146<br>p1275<br>38% Sc141	p1609<br>26 % Sc66
Streptococcus pneumoniae TIGR4 **Lactobacillales**	**Gram+**	p1566<br>26% Sc60<br>p1577<br>29% Sc46<br>p1232<br>26% Sc45	None	p2312<br>NeuA 99 % Sc1649<br>p1573<br>NeuB 99% Sc1424<br>p1231<br>NeuC 99% Sc1513	p1562<br>32%Sc154<br>p1234<br>31% Sc169	p1571<br>38% Sc142<br>p1235<br>41%Sc144	p1561<br>26% Sc66<br>p1229<br>NanK but not accessible by BLAST
Streptococcus pyogenes ATCC10782 **Lactobacillales**	**Gram+**	p1566<br>30% Sc61.6	None	None	p1564<br>31% Sc154	p1570<br>37% Sc 131	p1563<br>27% Sc 72.8

Species	Gram	YhcH *Escherichia coli* K12	NanM *Escherichia coli* K12 NanM *C. perfringens*	Sialidase NeuA /NeuB *Streptococcus agalactiae* 2603VR	NanA Aldolase *Escherichia coli* K12	NanE Epimerase *Escherichia coli* K12	NanK Kinase *Escherichia coli* K12
Clostridium perfringens ATCC 13124 **Clostridiales**	**Gram+**	p167 28 % Sc62	p695 27 % Sc93 100% Circularly permuted SP present	p694 NeuA 35% Sc347 p511 NeuA 32% Sc301	p165 37% Sc216	p164 41% Sc158	p168 24% sc72
Staphylococcus aureus 04-02981 **Bacillales**	**Gram+**		none	None	p318 37 % Sc215	p321 38%	p319 30 %
Ruminococcus gnavus AGR2154 **Clostridiales**	**Gram+**	p2363* 27% Sc47.8 Not visible in www.theseed.org	None	p2358 NeuB 42% Sc531	p2356 30% Sc150	p2357 39% Sc145	p2355 24% Sc59
Lactobacillus plantarum JDN1 **Lactobacillales**	**Gram+**	None	None	None	p2880 38% Sc200	p2881 40% Sc113	p2879 26% Sc42
Mycoplasma capricolum ATCC 27343 **Mycoplasmatales**	**Non Gram**	p413 23% Sc40	None	None	p411 36% Sc205	p415 37% Sc130	p414 22% Sc49

SI Reference

1. A. Teplyakov, G. Obmolova, J. Toedt, M. Y. Galperin, G. L. Gilliland, Crystal Structure of the Bacterial YhcH Protein Indicates a Role in Sialic Acid Catabolism. J. Bacteriol. 187, 5520–5527 (2005).
2. W. L. DeLano, Use of PYMOL as a communications tool for molecular science. Am. Chem. Soc. 228, 313–314 (2004).
3. F. Sievers, D. G. Higgins, Clustal Omega, accurate alignment of very large numbers of sequences. Methods Mol. Biol. Clifton NJ 1079, 105–116 (2014).
4. R. Overbeek, *et al*., The Subsystems Approach to Genome Annotation and its Use in the Project to Annotate 1000 Genomes. Nucleic Acids Res. 33, 5691–5702 (2005).
5. J. J. Almagro Armenteros, *et al*., SignalP 5.0 improves signal peptide predictions using deep neural networks. Nat. Biotechnol. 37, 420–423 (2019).
6. E. Severi, *et al.*, Sialic Acid Mutarotation Is Catalyzed by the *Escherichia coli* β-Propeller Protein YjhT. *J. Biol. Chem.* **283**, 4841–4849 (2008).
